# First isolation of *Mycobacterium saskatchewanense* from medical devices

**DOI:** 10.1038/s41598-023-48974-w

**Published:** 2023-12-07

**Authors:** Francesco Bisognin, Vincenzo Ferraro, Federica Sorella, Giulia Lombardi, Tiziana Lazzarotto, Paola Dal Monte

**Affiliations:** 1 Department of Medical Science and Surgery, University of Bologna, Bologna, Italy; 2 Microbiology Unit, IRCCS Azienda Ospedaliero Universitaria di Bologna, Bologna, Italy

**Keywords:** Bacteriology, Clinical microbiology, Microbiology

## Abstract

*Mycobacterium saskatchewanense* is a species of pigmented slow-growing Non-Tuberculous Mycobacteria (NTM), positive for *Mycobacterium avium* complex (MAC) by AccuProbe system. MAC organisms have frequently been isolated from different medical devices. This is the first study reporting isolation of *M. saskatchewanense* from medical devices and highlights the importance of correctly identifying the NTMs that often colonize sanitary water. GenoType Mycobacterium CM CE-IVD kit (CM) was used as the first step of NTM strain identification, and all positive cultures were found to be components of MAC. Then, GenoType NTM-DR CE-IVD kit (NTM-DR) was used to differentiate the different species. Sub-culture on solid media were used for: (i) phenotypical confirmation by colony morphology and *Matrix-Assisted Laser Desorption/Ionization-Time of Flight* (MALDI-TOF) mass spectrometry; (ii) molecular confirmation by Next Generation Sequencing. All positive cultures were identified as *M. intracellulare* by CM and NTM-DR assays, whereas colony morphology showed bright yellow scotochromogenic growth. MALDI-TOF analyses identified the strains as *M. saskatchewanense* with a high score, and identification was confirmed by NGS analysis based on the hsp-65 region. This paper suggests that it is important to actively monitor NTM contamination in medical devices that use sanitary water, to prevent the possibility of patients becoming infected.

## Introduction

Non-Tuberculous Mycobacteria (NTM) are a large and heterogeneous group of acid-fast bacilli that are widespread in the environment. Their cell walls contain a high proportion of fatty acids, making them highly hydrophobic. This enables mycobacteria to form biofilm and survive chemical treatments used to disinfect water, such as chlorine. For these reasons, NTM can cause water-borne infections, and healthcare facility water systems can be a reservoir for these microorganisms^1–4^.

*Mycobacterium Avium* Complex (MAC) organisms, including *M. avium, M. intracellulare and M. chimaera*, are the most frequent NTM causing chronic and disseminated disease in immune-compromised patients. MAC organisms have frequently been isolated from water samples, collected from different medical devices*.* In particular, in 2013 *M. chimaera* was identified as the causative agent of invasive infections in cardiothoracic surgery patients with extracorporeal circulation. In 2015 it emerged that the heater-cooler units (HCU) used during open-chest heart surgery were contaminated with *M. chimaera* causing infection by airborne transmission through ventilation fans^5,6^.

*M. saskatchewanense* has been identified as a new species of pigmented and slow-growing NTM, positive for *Mycobacterium avium* complex (MAC) by AccuProbe system^7^. Molecular assays based on DNA STRIP technology are the most commonly used tests to identify clinically relevant mycobacteria (approximately 30 species) from cultured material. However, these rapid assays may mis-identify NTM species. Currently, microbiology laboratories can identify most NTM species (approximately 180 species) either through mass spectrometry, which often requires a subculture on solid media in order to obtain a high level of confidence, or through NGS analysis requiring a specialized technician and expensive equipment. This study describes the first isolation of *M. saskatchewanense* from medical devices and highlights the importance of correctly identifying NTMs that often colonize sanitary water.

## Results

**Figure 1:**
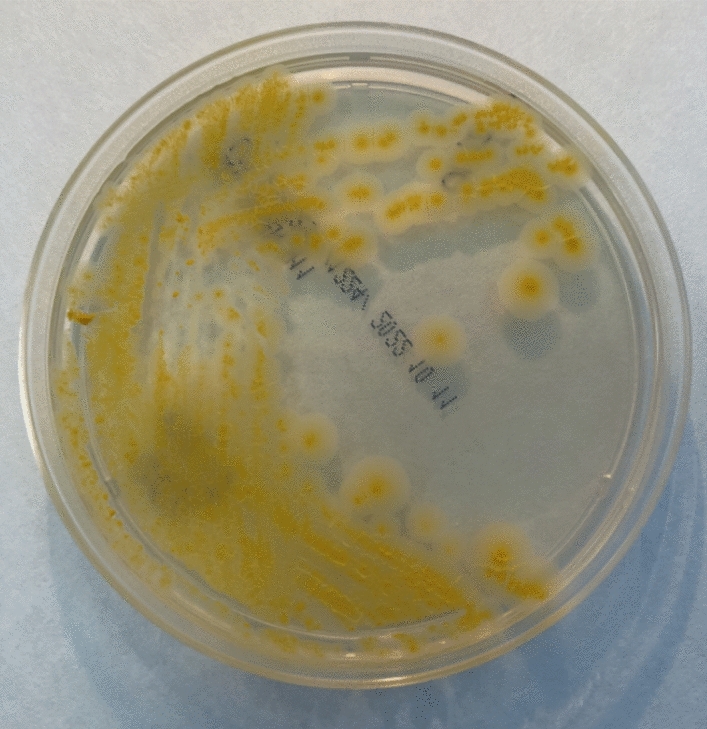
Colony morphology of *M. saskatchewanense*. This NTM appears as a bright yellow scotochromogenic colony on Middlebrook 7H11 agar.

Sixteen (13%) out of 112 dialysis fluid MGIT cultures were positive for NTM, verified by acid-fast bacilli staining. All positive cultures were identified as *M. intracellulare* by Genotype CM and NTM-DR molecular assays. However, sub-cultures on Middlebrook 7H11 agar did not produce the colony morphology expected for *M. intracellulare,* as bright yellow scotochromogenic growth was observed (Fig. 1). Further analyses performed by *Matrix-Assisted Laser Desorption/Ionization-Time of Flight*” (MALDI-TOF) mass spectrometry identified all the strains as *M. saskatchewanense* with a high score (> 1.8).

**Figure 2:**
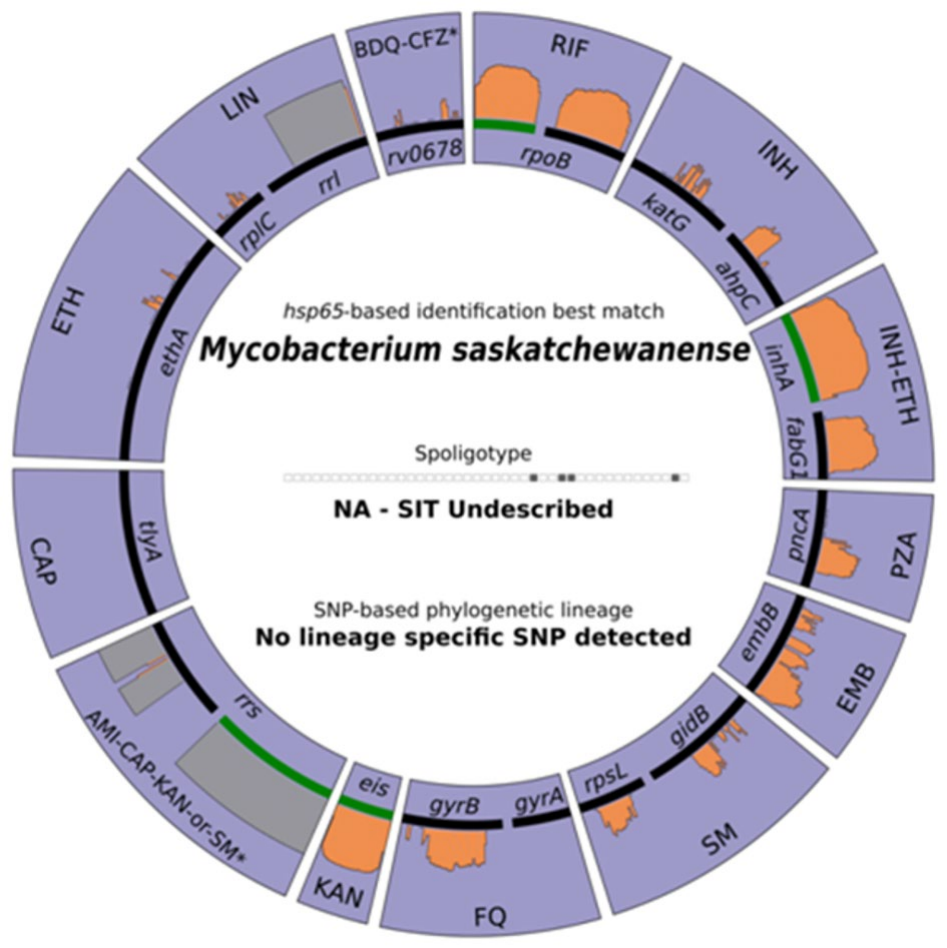
Output of Deeplex Myc-TB test. Identification of *M. saskatchewanense* by hsp65-based sequencing.

The result obtained by hsp65-based sequencing with Deeplex Myc-TB test, confirmed the identity of the bacterial isolates as *M. saskatchewanense* (Fig. 2).

## Discussion

Nontuberculous mycobacteria (NTM) are frequently the cause of opportunistic infection in immunocompromised hosts. Their ability to form biofilm and withstand chemical treatments has caused them to emerge as opportunistic pathogens within healthcare facilities^1^.

Numerous studies have reported elevated concentrations of mycobacteria in water systems within healthcare facilities^8–10^*.* Plumbing systems of large structures often have dead legs and dead ends, which may harbour biofilms, potentially including NTM. Furthermore, there is substantial evidence that the zinc in galvanized plumbing may contribute to the persistence of MAC organisms in these distribution systems^11^.

Waterborne infections from nontuberculous mycobacteria in healthcare facility water systems have been systematically reviewed. The most commonly affected patient populations were immunocompromised, post-surgical, and haemodialysis. The main routes of exposure included central venous catheters (CVCs), wound exposure, and contamination during surgery^1,4^.

Contamination of medical devices or aqueous solutions is often implicated in the transmission of infection. Recently, the slow grower *M. chimaera* has been reported in the setting of contaminated heater-cooler devices used in open-heart surgery and extracorporeal membrane oxygenation procedures^5,12^.

Our previous research showed that *M. chimaera* subtypes circulating in hospital plumbing systems passed through absolute filters, highlighting the possibility that other medical equipment using sanitized water, such as endoscope reprocessing devices or haemodialysis systems, could also become contaminated by NTM^13^.

So far in haemodialysis patients a few outbreaks have been reported, due to contamination by NTM stains belonging to *M. chelonae* and *to M. abscessus complex* and inadequate disinfection of disposable high-flux hollow-firer dialyzers^14^. In contrast, peritonitis caused by NTM is an important complication in peritoneal dialysis patients. According to the review by Song et al*.*^15^, over half of the peritonitis cases could be attributed to rapidly growing *Mycobacterium* such as *M. fortuitum* (38.6%) and *M. chelonae* (14.0%). The prevalence, risk factors, and mortality of NTM infections in patients with end-stage renal disease have recently been examined, revealing an increased risk of mortality in cases involving NTM diagnosis^16^.

*M. saskatchewanense* was first identified in 2004, as a new species of pigmented slow-growing NTM, positive for *Mycobacterium avium* complex (MAC) by AccuProbe system from sputum and pleural fluid of a patient with bronchiectasis^7^. A further report associated *M. saskatchewanense* with a chronic kidney disease patient, who required dialysis for 2 years prior to evaluation for renal transplant^17^.

This is the first paper where *M. saskatchewanense* has been isolated from a large number of ultrapure dialysis fluid water samples.

Differentiation of mycobacteria to the species level by genotypic and phenotypic assay sometimes leads to inaccurate results. Here we compared the identification methods commonly used in mycobacteriology laboratories: DNA STRIP technology, MALDI-TOF mass spectrometry, and hsp65-based sequencing.

The correct identification of *M. saskatchewanense* was obtained by MALDI-TOF mass spectrometry with a high score, while DNA STRIP technology, the most commonly used method for NTM identification, misidentified it as *M. intracellulare.* Culture remains essential for NTM isolation and colony morphology can support identification. Therefore, laboratories that use DNA STRIP technology for NTM identification must use a confirmatory method to achieve correct classification.

This paper suggests that it is important to actively monitor NTM contamination in medical devices that use sanitary water, to prevent the possibility of patients becoming infected.

## Methods

From August to November 2022, 112 ultrapure dialysis fluid samples from dialysis machines were processed by the referral centre for the detection of mycobacteria from environmental specimens in the Emilia-Romagna Region, at the Microbiology Unit, IRCCS University Hospital of Bologna, Italy.

Each water sample (1 L) was filtered with cellulose nitrate membranes (0.45 µm), using Microsart filtration system (Sartorius, Germany) according to ECDC guidelines and the residue resuspended in 10 mL of 0.9% saline solution^12^. Concentrated samples were treated as previously described^13^. Samples were decontaminated using BBL MycoPrep solution (Becton Dickinson, USA), resuspended in 2 mL of phosphate buffered solution, inoculated onto solid medium (Lowenstein Jensen, Heipha Diagnostics, Germany) and into Middlebrook 7H9 Broth (MGIT, Becton Dickinson, USA) and cultured for 42 days. Ziehl–Neelsen staining was used to confirm mycobacterial presence in positive cultures.

Mycobacteria from positive MGIT cultures were identified as NTM by DNA STRIP technology using first Genotype CM CE-IVD kit (CM, Bruker, Germany) and then, if belonging to *Mycobacterium avium* complex, analysed by Genotype NTM-DR CE-IVD kit (NTM-DR, Bruker, Germany). Positive MGIT were sub-cultured in Middlebrook 7H11 Agar (7H11 plate, Becton Dickinson, USA) for approximately 2 weeks to obtain microbial growth for phenotypic confirmation by morphological features and MALDI-TOF mass spectrometry (Bruker, Germany). A small amount of bacterial biomass, picked from 7H11 plates, was extracted by adding 50 µL of pure acetonitrile, 50 µL of 70% formic acid and vortexing. Then, the solution was centrifuged and 1 μl of supernatant was spotted on a MALDI target, and allowed to air dry before MALDI-TOF analysis.

Bacterial strains were subsequently sequenced with Deeplex Myc-TB CE-IVD kit (GenoScreen, France) according to the manifacturer’s instructions, to confirm identification.

## Data Availability

The dataset used during the current study is available from the corresponding author on reasonable request.
